# Genome-based population structure analysis of the strawberry plant pathogen *Xanthomonas fragariae* reveals two distinct groups that evolved independently before its species description

**DOI:** 10.1099/mgen.0.000189

**Published:** 2018-06-06

**Authors:** Michael Gétaz, Marjon Krijger, Fabio Rezzonico, Theo H. M. Smits, Jan M. van der Wolf, Joël F. Pothier

**Affiliations:** ^1^​ Environmental Genomics and Systems Biology Research Group, Institute of Natural Resource Sciences, Zurich University of Applied Sciences (ZHAW), CH-8820 Wädenswil, Switzerland; ^2^​ Wageningen University and Research, Wageningen, the Netherlands

**Keywords:** MLVA, VNTR, CRISPR, bacterial disease, angular leaf spots, MLSA

## Abstract

*Xanthomonas fragariae* is a quarantine organism in Europe, causing angular leaf spots on strawberry plants. It is spreading worldwide in strawberry-producing regions due to import of plant material through trade and human activities. In order to resolve the population structure at the strain level, we have employed high-resolution molecular typing tools on a comprehensive strain collection representing global and temporal distribution of the pathogen. Clustered regularly interspaced short palindromic repeat regions (CRISPRs) and variable number of tandem repeats (VNTRs) were identified within the reference genome of *X. fragariae* LMG 25863 as a potential source of variation. Strains from our collection were whole-genome sequenced and used in order to identify variable spacers and repeats for discriminative purpose. CRISPR spacer analysis and multiple-locus VNTR analysis (MLVA) displayed a congruent population structure, in which two major groups and a total of four subgroups were revealed. The two main groups were genetically separated before the first *X. fragariae* isolate was described and are potentially responsible for the worldwide expansion of the bacterial disease. Three primer sets were designed for discriminating CRISPR-associated markers in order to streamline group determination of novel isolates. Overall, this study describes typing methods to discriminate strains and monitor the pathogen population structure, more especially in the view of a new outbreak of the pathogen.

## Data Summary

All genome sequencing data have been deposited in EMBL under the study code PRJEB25730 (https://www.ebi.ac.uk/ena/data/view/PRJEB25730) with the accession numbers ERR2528852–ERR2528906 for raw reads (https://www.ebi.ac.uk/ena/data/view/ERR2528852-ERR2528906) and ORXX01000000–ORZZ01000000 for genome assemblies (https://www.ebi.ac.uk/ena/data/view/ORXX01000000-ORZZ01000000).

**Impact Statement**

The population structure analysis of *Xanthomonas fragariae* was performed using two marker types: VNTRs (MLVA) and CRISPR spacers. The congruent results of both marker analyses yielded complementary results for an enhanced understanding of the evolutionary history of the pathogens since they were first reported in 1960. A CRISPR system harbouring differential spacer sequences allowed the identification of four (sub)groups. The main groups were already separated in 1960, indicating that strains may have coexisted and evolved separately until nowadays. Three sets of primers were designed, based on discriminative markers, to streamline the characterization of novel isolates in order to refine the population structure obtained in this study. The bacterial disease has been lately reported from new countries, which proves the continuous spread of the disease and the urgency to investigate the evolution and propagation of this bacterial disease. The described technique is likely to be utilized for tracking and tracing novel isolates in the framework of *X. fragariae* eradication programs.

## Introduction

Assessing evolutionary changes within closely related microbial isolates by epidemiological typing requires the availability of molecular markers that differentiate isolates within a species [1]. The evolution of genomes is related to various factors and different molecular typing targets have diverse ‘molecular clock speeds’ [1]. The use of variable number of tandem repeats (VNTRs) has been found to be an efficient genotyping method in bacteria, as they provide a high level of discriminatory power for strain differentiation because of their high mutability [1, 2]. The variation of the repeats is caused by recombination-mediated events [3] and slipped-strand mispairing (SSM), which is produced between mother and daughter strand during DNA replication, resulting in a change in the number of unit repeats [4, 5]. The analysis of VNTR variation, also called multiple-locus VNTR analysis (MLVA), was utilized at first to study population structure of human pathogens [6, 7]. Subsequently, geographical information can be added to genotype information in order to study how specific genetic variants of the pathogen behave in terms of geographic representation or outbreaks [8, 9]. This approach has been used successfully for various plant-pathogenic bacterial species [2, 10] including for global surveillance of the plant pathogens *Xanthomonas citri* pv. *citri* [11] and *Xanthomonas oryzae* [12].

Clustered regularly interspaced short palindromic repeat regions (CRISPRs) are arrays of conserved DNA repeats that can vary between 24 and 48 bp, which are interspaced by unique and similarly-sized spacers [13–15]. Typically, the CRISPR system includes a leader sequence [14], which is thought to play a role as a promoter to transcribe the CRISPR array [16], directly adjacent to the first repeat. Another element enclosed in the CRISPR system are *cas* (CRISPR-associated) genes [17], which encode proteins containing endonuclease and exonuclease activities, helicases, RNA- and DNA-binding motifs and proteins involved in transcriptional regulation [14, 17]. It has been shown that the CRISPR/*cas* system provides heritable acquired resistance against phages in some prokaryotes [18] and limits horizontal gene transfer of plasmids [19]. The genetic information can be used for evolutionary purposes, since insertions in the CRISPR repeat region (CRR) take place in a polar manner next to the leader sequence, thus making it possible to assign a sequential order to the acquired spacers [20]. Similarly, subsequent spacer deletions within the sequence can also be exploited to infer a chronological order between different isolates sharing similar CRRs [20, 21]. The record of past encounters with foreign DNA included in the CRISPR spacer is chronological and may be correlated to a geographical component, since spatially distant isolates are normally exposed to different backgrounds of mobile DNA sequences [22]. The CRISPR sequences have been described as one of the most rapid evolving elements in a bacterial genome, whereby highly identical strains (more than 99 % identity at genome level), were found to differ significantly only in their CRR sequences [20, 23, 24]. The CRISPR/*cas* system has already been analysed in several plant pathogenic bacteria for diversity, evolution of strains and epidemiological analyses [22, 25, 26].

Angular leaf spots (ALS) on strawberry plants are caused by the Gram-negative gammaproteobacterium *Xanthomonas fragariae* [27] that is categorized as a quarantine organism by the European and Mediterranean Plant Protection Organization [28]. The bacterium has been reported to be the major bacterial disease affecting cultivated strawberry (*Fragaria*×*ananassa* Duchesne) [29], and has become increasingly problematic in agricultural strawberry production [30]. The disease was first described in 1960 in Minnesota, USA [27] and then formally reported in most major strawberry-producing regions worldwide [31, 32]. Long distance spread is mainly due to international transport of plant material through human trade [33]. Spread often happens via asymptomatic strawberry plants, when symptoms are not detectable by visual inspection at the moment of the plant material exportation [34]. In the early infection stage, symptoms include water-soaked foliar lesions, appearing as light green spots, which are transparent when viewed with transmitted light [27, 35]. The bacterial infection generally does not result in host plant mortality. Lesions often become necrotic, resulting in reduction in total yield [36, 37]. Losses due to reduced marketable yield have been reported for strawberry fields, i.e. 8 % from experimental plots in 1995 in the USA [37], 10 to 30 % in Germany [38], or even 75 to 80 % yield loss from an irrigated plot in the USA in the 1960s [39].

A better understanding of bacterial evolutionary history and geographic dispersal is essential to help with identifying inoculum sources in order to prevent future spread. Knowledge on the genetic diversity among *X. fragariae* strains and population dynamics allows the design of an efficient strategy for disease management and control [40], reducing the chance of economic losses due to the disease. Using a combination of PCR methods [41], RFLP [42] and AFLP [43] it has been previously shown that between three and nine subgroups of *X. fragariae* strains may exist, but dispersal pathways and the influence of the geographic origin were not studied in detail. In this study, *X. fragariae* genomic sequences were screened for the presence of both CRISPR and VNTR markers. Both genotyping methods were able to separate isolates into two main groups and a total of four subgroups. Additionally, an easy-to-perform typing scheme was designed to classify novel isolates into the obtained (sub)groups as a tool to allow source-tracking of *X. fragariae*.

## Methods

### Bacterial strains

A total of 58 bacterial strains of *X. fragariae* obtained from public and private collections (Table 1) were used in this study. Strains were selected in order to obtain a collection with various years of isolation and geographical origins. Strain identity was confirmed using the *X. fragariae*-specific loop-mediated isothermal amplification (LAMP) assay [44]. The draft genomes of LMG 25863 (accession number: AJRZ01000000) [45] and of two American isolates, *Fa*P21 (accession numbers: CP016830–CP016832) and *Fa*P29 (accession numbers: CP016833–CP016835) [46] were obtained from GenBank and were added to the *in silico* analysis.

**Table 1.** *Xanthomonas fragariae* strains used in the development of VNTR and CRISPR analysis, their geographic origin, host of isolation, year of isolation, MLVA profiles, CRISPR types and CRISPR spacer composition

Strain*	**Geographic origin†**	**Host plant**	**Year of isolation**	**MLVA types‡**	**CRISPR types§**	**CRISPR spacer sequence||**
PD 885^T^	US	*Fragaria chiloensis* var. ananassa	1960	III	*Xf*-CGr-IA	A
PD 4314	US	*Fragaria chiloensis* var. ananassa	1960	III	*Xf*-CGr-IA	A
PD 2659	US	*Fragaria* sp.	1962	III	*Xf*-CGr-IA	A
ICMP 661	US	*Fragaria* sp.	1982	III	*Xf*-CGr-IA	A
ICMP 659	US	*Fragaria* sp.	1982	III	*Xf*-CGr-IA	A
XF 61	NL	*Fragaria* sp. Figaro	2010	II	*Xf*-CGr-IB	B
JVD-0051	BE	*Fragaria*×*ananassa*	2002	II	*Xf*-CGr-IB	A
PD 2905	PT	*Fragaria* sp. Cambridge vigour	1996	II	*Xf*-CGr-IB	B
CFBP 4784	PT	*Fragaria* sp. Thulda.	1995	IV	*Xf*-CGr-IC	A
ICMP 13779	IT	*Fragaria*×*ananassa* Duchesne	1996	IV	*Xf*-CGr-IC	A
ICMP 13780	IT	*Fragaria*×*ananassa* Duchesne	1996	IV	*Xf*-CGr-IC	B
PD 3170	NL	*Fragaria* sp.	1996	IV	*Xf*-CGr-IC	A
CFBP 4785	PT	*Fragaria* sp.	1998	IV	*Xf-*CGr-IC	A
CFBP 4786	PT	*Fragaria* sp.	1998	IV	*Xf*-CGr-IC	C
CFBP 5253	FR	*Fragaria* sp.	1999	IV	*Xf*-CGr-IC	A
CFBP 5257	FR	*Fragaria* sp.	1999	IV	*Xf*-CGr-IC	A
CFBP 5260	FR	*Fragaria* sp.	1999	IV	*Xf*-CGr-IC	A
PD 4634	NL	*Fragaria* sp.	2003	IV	*Xf*-CGr-IC	A
PD 4932	NL	*Fragaria* sp. Elsanta	2004	IV	*Xf*-CGr-IC	A
PD 5205	NL	*Fragaria* sp. Elsanta	2005	IV	*Xf*-CGr-IC	A
PD 5365	NL	*Fragaria* sp. Elsanta	2006	IV	*Xf*-CGr-IC	A
PD 5482	NL	*Fragaria* sp. Figaro	2007	IV	*Xf*-CGr-IC	A
PD 6879	NL	*Fragaria* sp.	2008	IV	*Xf*-CGr-IC	A
PD 6840	NL	*Fragaria* sp.	2009	IV	*Xf*-CGr-IC	A
LMG 25863	BE	*Fragaria*×*ananassa* Elsanta	2011	IV	*Xf*-CGr-IC	A
PD 6880	NL	*Fragaria* sp. Elsanta	2011	IV	*Xf*-CGr-IC	A
*Fa*P29	US	*Fragaria*×*ananassa* Portola	2011	IV	*Xf*-CGr-IC	A
*Fa*P21	US	*Fragaria*×*ananassa* Portola	2011	IV	*Xf*-CGr-IC	A
PD 6881	NL	*Fragaria* sp. Elsanta	2012	IV	*Xf*-CGr-IC	A
PD 6882	NL	*Fragaria* sp. Elsanta	2013	IV	*Xf*-CGr-IC	A
ICMP 20572	US	*Fragaria*×*ananassa* Duchesne	2014	IV	*Xf*-CGr-IC	A
ICMP 20573	US	*Fragaria*×*ananassa* Duchesne	2014	IV	*Xf*-CGr-IC	A
ICMP 20574	US	*Fragaria*×*ananassa* Duchesne	2014	IV	*Xf*-CGr-IC	E
ICMP 20575	US	*Fragaria*×*ananassa* Duchesne	2014	IV	*Xf*-CGr-IC	A
GBBC 2087	BE	*Fragaria*×*ananassa*	1999	IV	*Xf*-CGr-IC	A
JVD-0046	BE	*Fragaria*×*ananassa*	2002	IV	*Xf*-CGr-IC	C
JVD-0047	BE	*Fragaria*×*ananassa*	2002	IV	*Xf*-CGr-IC	A
GBBC 2088	BE	*Fragaria*×*ananassa*	2000	IV	*Xf-*CGr-IC	D
NCPPB 1822	US	*Fragaria chiloensis* var. ananassa	1966	I	*Xf*-CGr-II	A
ICMP 6269	NZ	*Fragaria*×*ananassa* Duchesne	1971	I	*Xf-*CGr-II	A
NCPPB 2473	IT	*Fragaria vesca*	1972	I	*Xf*-CGr-II	A
PD 2660	NZ	*Fragaria* sp. Tioga	1972	I	*Xf*-CGr-II	A
ICMP 6277	NZ	*Fragaria*×*ananassa* Duchesne	1972	I	*Xf*-CGr-II	A
ICMP 6278	NZ	*Fragaria*×*ananassa* Duchesne	1972	I	*Xf*-CGr-II	A
CFBP 1558	FR	*Fragaria* sp.	1974	I	*Xf*-CGr-II	A
ICMP 4019	NZ	*Fragaria*×*ananassa* Duchesne	1974	I	*Xf-*CGr-II	A
CFBP 6767	AU	*Fragaria*×*ananassa*	1975	I	*Xf-*CGr-II	A
ICMP 6646	AU	*Fragaria*×*ananassa* Duchesne	1975	I	*Xf-*CGr-II	A
ICMP 6648	AU	*Fragaria*×*ananassa* Duchesne	1975	I	*Xf*-CGr-II	A
ICMP 5813	BR	*Fragaria*×*ananassa* Duchesne	1977	I	*Xf*-CGr-II	B
PD 2662	GR	*Fragaria* sp.	1979	I	*Xf-*CGr-II	A
CFBP 2510	ES	*Fragaria* sp.	1982	I	*Xf-*CGr-II	A
NCPPB 3743	BR	*Fragaria* hybrid cv. Campinas	1990	I	*Xf-*CGr-II	B
F1-81	CH	*Fragaria* sp.	1993	I	*Xf-*CGr-II	A
F1-80	CH	*Fragaria* cv. Polka	1993	I	*Xf-*CGr-II	A
F2-02	CH	*Fragaria* sp.	1994	I	*Xf-*CGr-II	A
PD 3145	ES	*Fragaria* sp.	1997	I	*Xf*-CGr-II	A
NBC 2815	ES	*Fragaria* sp.	1997	I	*Xf*-CGr-II	A

*Culture collection providing strains are abbreviated in the strain names as CFBP (Collection Française de Bactéries associées aux Plantes, France), ICMP (International Collection of Microorganisms from Plants, New Zealand), GBBC (culture collection of the Diagnostic Centre for Plants of ILVO, Belgium), LMG (Collection of the Laboratorium voor Microbiologie en Microbiele Genetica, Belgium), NCPPB (National Collection of Plant Pathogenic Bacteria, United Kingdom), PD (Culture Collection of Plant Pathogenic Bacteria, the Netherlands) and NBC (Naktuinbouw Bacterial Collection, the Netherlands).

†AU, Australia; BE, Belgium; BR, Brazil; CH, Switzerland; ES, Spain; FR, France; GR, Greece; IT, Italy; NL, The Netherlands; NZ, New Zealand; PT, Portugal; US, United States of America.

‡MLVA profile as determined in this study using 36 loci.

§CRISPR group as determined in this study using spacer variations of the only variable CRISPR array (*Xf*-CRR-2).

*||*CRISPR spacer sequence as described in Fig. 2 with different sequence of spacer within a given CRISPR type.

### DNA sequencing and assembling

Whole-genome sequencing was chosen for this study in order to access the VNTR and CRISPR information. For this, genomic DNA of 20 isolates belonging to the private collection in Wageningen (the Netherlands) was extracted with the Wizard Magnetic DNA Purification System for Food (Promega) according to the instruction of manufacturer. Subsequently, libraries were prepared with the Nextera DNA Flex kit (Illumina) and were sequenced using a HiSeq 2000 sequencer (Illumina) with paired 100 bp read length. Additionally, the DNA of 35 strains was extracted with a standard protocol for cultured cells in the NucleoSpin Tissue Kit (Macherey–Nagel). Libraries were prepared with the Nextera XT DNA kit (Illumina) and sequenced on a MiSeq sequencer (Illumina), generating paired 300 bp read lengths. Raw data for all genomes were *de novo* assembled with SeqMan NGen v.12.1.0 software (dnastar). Three genomes sequenced with HiSeq were sequenced as well with PacBio technology in order to obtain complete genome sequences (accession numbers: LT853880–LT853887) [47]. Data from the latter sequencing were used in this study.

### Marker screening

The markers used in this study were predicted using the first publicly available draft genome of *X. fragariae* LMG 25863 [45]. VNTRs were identified using the tandem repeat finder program JSTRING [48] using the default parameters (score=200, maxgamma=50, mild penalty). CRISPR repeat regions (CRRs) were identified using the web tool CRISPRfinder [49].

All sequenced genomes were screened for the presence of the regions containing VNTRs by including a 50 bp flanking sequence with BLASTn v.2.3.0+ [50] against the local database. For each strain, the number of repeats was determined. For CRR determination, all sequenced genomes were screened through CRISPRfinder [49]. CRISPR spacer sequences were extracted, and compared with each other with BLASTn v.2.3.0+ [50]. Matching spacers were aligned and assigned to a position number.

For further analyses, only the VNTRs and CRISPRs showing variations among the strains were considered. Due to the highly repetitive sequence within VNTRs, the risk of genome mis-assembling for markers that are longer than the read length generated by the sequencing technology is considerable. In this study, only two VNTR markers resulted from a longer fragment than 100 bp, which is the minimal read length for strains sequenced with the HiSeq technology. All markers were publicly stored in an MLVABank (http://microbesgenotyping.i2bc.paris-saclay.fr/databases/view/843).

### Analysis of global *X. fragariae* diversity

A first assessment of the bacterial diversity on our *X. fragariae* collection (Table 1) was obtained with the phylogenetic distance of seven partial sequences of housekeeping genes (*atpD*, *dnaK*, *etp*, *fyuA*, *glnA*, *gyrB* and *rpoD*; totalling approximately 6200 bp), which had been used previously for *Xanthomonas* MLSA studies [51, 52]. Concatenated sequences of partial genes were analysed with maximum likelihood with 1000 bootstraps to reconstruct a phylogenetic tree using mega v.6 [53]. The average nucleotide identities (ANIb) [54] were calculated on the genomes using PYANI v.0.2.0 [55] in order to determine the genetic similarity between strains.

The CRISPR leader sequence and *cas* gene region were compared with BLASTn v.2.3.0+ [50] in order to assess the divergence of the region in individual strains. CRISPR spacers were compared with a database of sequenced *X. fragariae* strains, as well as the nucleotide collection (nr/nt) on NCBI. VNTRs repeat numbers were processed with a temporary evaluation license of BioNumerics v.7.6 (Applied Maths) for a multiple-locus VNTR analysis (MLVA) in order to create minimal spanning trees.

### Design and validation of PCR primer sets

Each of the four (sub)groups established in this study displayed characteristic CRISPR regions composed of specific spacer sequences. Three PCR primer sets were designed in order to assign novel isolates to the four obtained *X. fragariae* CRISPR (sub)groups on the basis of the results of the PCR reactions. CRISPR repeats were chosen for the discrimination as differences based on a single repeat plus a spacer are in the order of 60 bp, as the resolution of a VNTR variation would not always allow the visualization of the variation on an agarose gel. Primer design was performed on at least one strain per group in Lasergene SeqBuilder v.12.1.0 (dnastar) and tested *in silico* with FastPCR v.6.1.1 beta 2 (PrimerDigital). Primers were subsequently synthesized by Microsynth (Balgach, Switzerland).

PCR validation was performed on boiled cells of all available strains of *X. fragariae* (Table 1). Strains were grown on agar plates with Wilbrinks-N medium [56] for 72 h. A full loop of bacteria was transferred to a 2 ml tube with 500 µl distilled water, boiled for 15 min at 95 °C and diluted tenfold in distilled water. Prior to PCR, verification with the LAMP detection system specific to *X. fragariae* [44] was applied to all samples to confirm them as being *X. fragariae* samples. This control is crucial in the eventuality of negative amplifications of a sample with all three primer sets, which could be caused by the occurrence of a deletion affecting one of more spacers targeted by primers.

Amplification was performed in a 20 µl reaction volume and consisting of 5 µl nuclease free water, 10 µl 2× KAPA2G Robust HotStart polymerase (KapaBiosystems), 0.5 µM forward primer, 0.5 µM reverse primer and 3 µl diluted bacterial boiled cells. Amplification was performed using a Bio-Rad PCR machine, with a thermal cycle programmed for 3 min at 95 °C as initial denaturation, followed by 35 cycles of 15 s at 95 °C for denaturation, 15 s at 60 °C as annealing, 15 s at 72 °C for extension, and 1 min at 72 °C for final extension. PCR products of four samples (PD 885^T^, PD 2905, PD 5205 and NBC 2815) belonging to the four described CRISPR (sub)groups were tested with a Fragment Analyzer (Advanced Analytical), with dsDNA 915 Reagent Kit (Advanced Analytical) and used as reference for band sizes and presence/absence per primer set. PCR products (3 µl) of all tested samples were examined through gel electrophoresis on a 1.5 % (w/v) agarose gel and scored under UV illumination.

## Results

### Bacterial collection sequenced with MiSeq and HiSeq technologies

A total of 55 strains of *X. fragariae* (Table 1) were sequenced in this study in order to access a panel of molecular markers to determine genetic diversity within the species. The number of contigs for strains sequenced with HiSeq technology varied between 269 and 321, whereas MiSeq sequencing yielded 258 to 455 contigs per strain (Table S1, available in the online version of this article). HiSeq assemblies had an average size of 3.85 Mb whereas MiSeq assemblies yielded genome sizes of approximately 4.20 Mb, which is the average size reached by the first *X. fragariae* draft genome [45] and more recently by complete genomes [46, 47]. Mapping of the assemblies from three strains sequenced with both HiSeq and PacBio technologies revealed that contig ends largely represented highly repetitive regions and transposable elements, making it impossible for genome assemblers to close these gaps using the reads from the short-insert technologies.

### Evaluation of genetic and genomic diversity using taxonomic markers

Seven housekeeping genes were used to analyse the intraspecies variability in *X. fragariae* by aligning the same partial gene sequences used in previous MLSA with species of the genus *Xanthomonas*. The results of analysis performed with mega v.6 [53] indicated that the species *X. fragariae* can be divided into two groups with a low phylogenetic distance (five SNPs within a total of 6206 bp, i.e. 0.01 % sequence divergence; Fig. S1). These results confirmed that *X. fragariae* forms a closely related group [41]. The average nucleotide identities (ANIb) using the genomes determined in this study confirmed that all strains represented the same species with values ranging between 99.48 and 99.97% identity (Table S2).

### *In silico* CRISPR prediction and marker screening for *X. fragariae* diversity

**Fig. 1.**
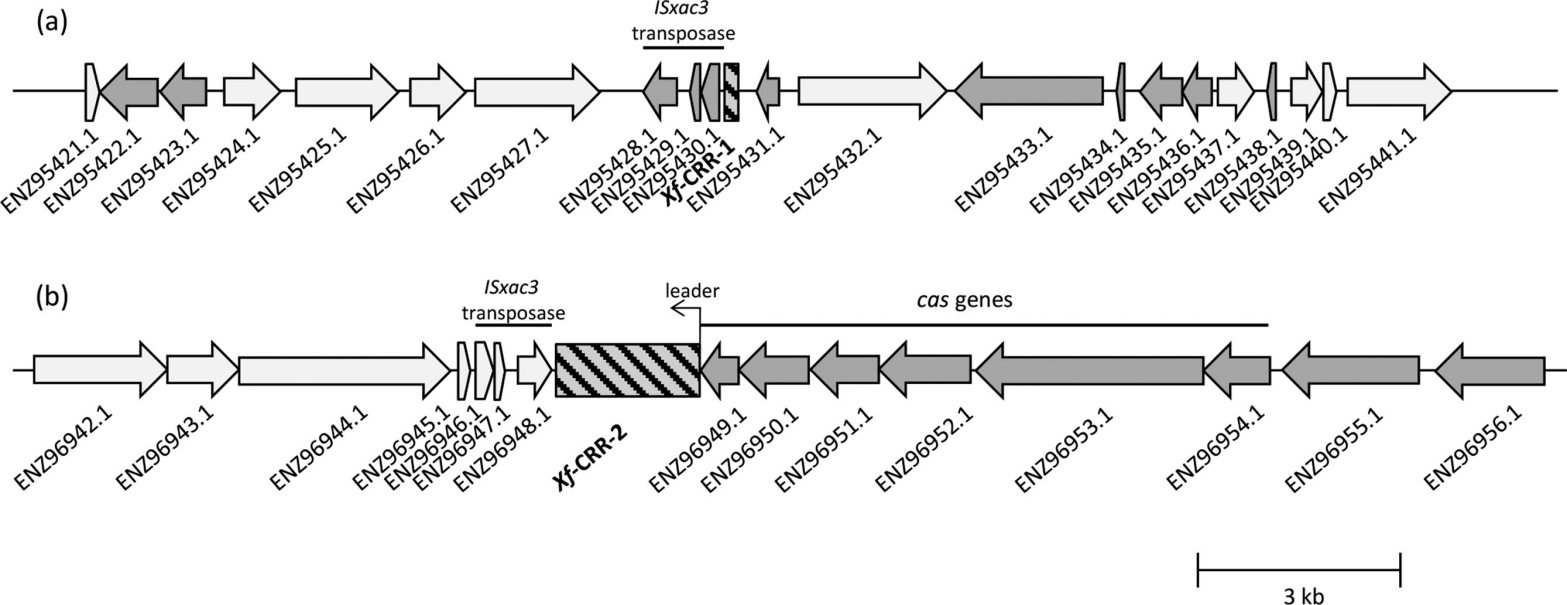
CRISPR regions in *Xanthomonas fragariae* LMG 25863 (GenBank WGS accession prefix AJRZ01), (a) *Xf*-CRR-1 located on contig 66 and (b) *Xf*-CRR-2 located on contig 10. The *cas* genes were located directly adjoining *Xf*-CRR-2. Both CRISPR repeat regions, which are represented by hatched rectangles, are located beside an identical insertion sequence element *ISxac3*.

Two CRRs were identified in the *X. fragariae* LMG 25863 genome data, which were named *Xf*-CRR-1 and *Xf*-CRR-2 (Fig. 1). In this strain, *Xf*-CRR-1 contains only three spacers of 32 bp whereas 35 spacers of 32 bp were identified in *Xf*-CRR-2. Both CRRs displayed an identical 28 bp direct repeat (DR). The distance estimated between the two CRRs on complete genomes [47] was approximately 250 kb, and both *Xf*-CRR-1 and *Xf*-CRR-2 were located directly adjacent to identical *ISxac*3 transposase region (Fig. 1). Both *Xf*-CRR were probably forming a unique CRR, which was split into two regions by the transposase. Subsequently, *Xf*-CRR-1 may have been transposed to another region far from *cas* genes, which may explain its observed inactivity. This same distant configuration between the two *Xf*-CRR was confirmed in the genomes harbouring one full chromosome [46, 47].

**Fig. 2.**
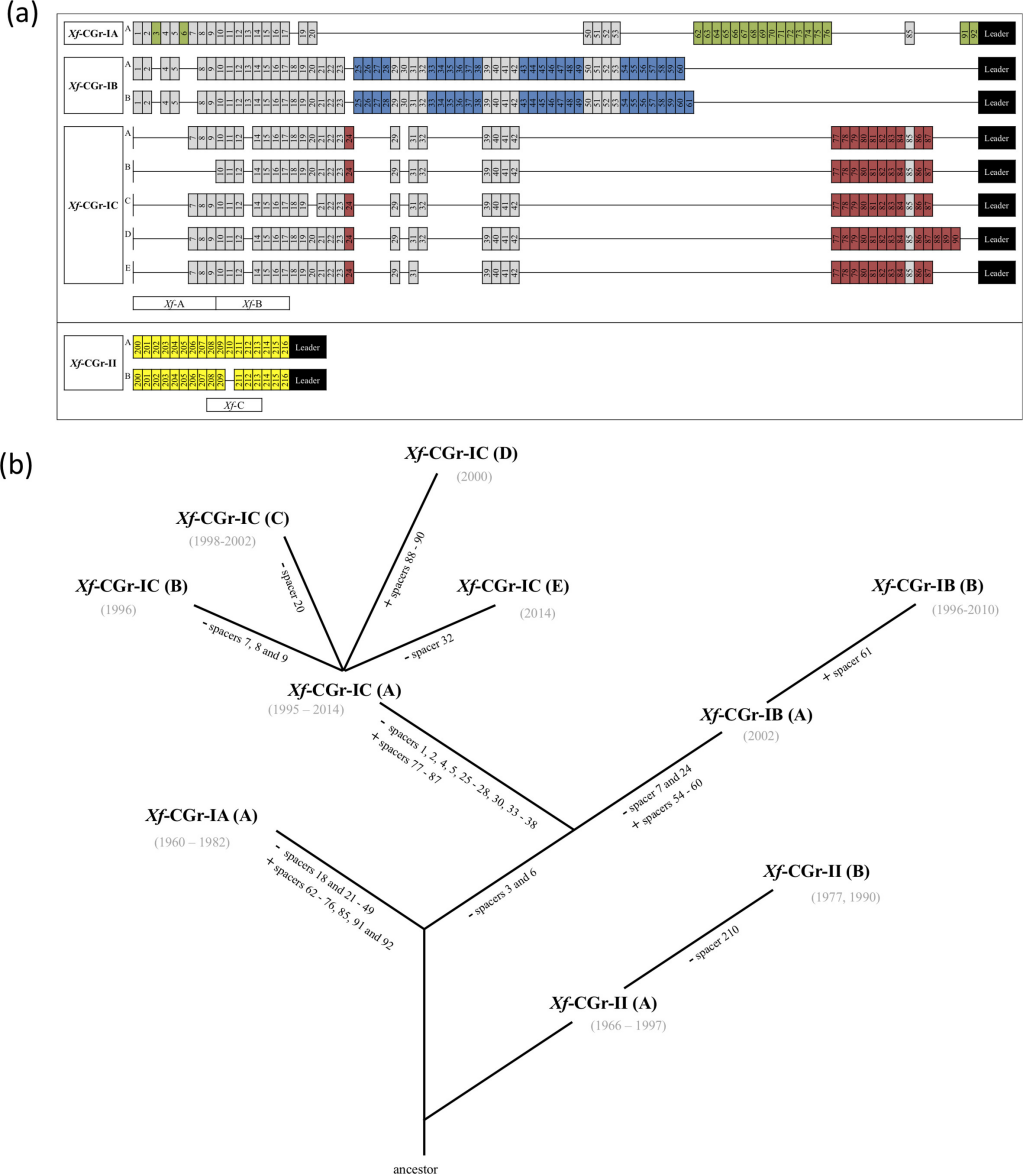
(a) CRISPR repeat regions identified in *Xanthomonas fragariae* (sub)groups. The leader sequence is indicated at the right. A different numbering system was used between groups *Xf*-CGr-I (1 to 92) and -II (200 to 216). Grey boxes represent the spacers found in more than one group, whereas coloured boxes represent spacers that were specific to a given group. In individual CRISPR (sub)groups, different CRISPR spacer sequence were observed and were named alphabetically from A to E. PCR amplicons designed on the basis of CRISPR spacers are represented by boxes and indicated under repeats per group and are labelled *Xf*-A, *Xf*-B and *Xf*-C. (b) Hypothesis for an evolutionary relationship between *X. fragariae* (sub)groups inferred from the acquisition (+) or loss (−) of CRISPR spacers. The years of isolations of the strains belonging to each subgroup are listed in grey under the subgroup.

The *X. fragariae* genomes (Table S1) were screened for CRR presence and variability among strains. The screening revealed that only *Xf*-CRR-2 was variable in terms of spacer composition and could thus be used for further analysis (Fig. 2; Table S3). The leader sequence of *Xf*-CRR-2 was identical for all strains. All *cas* genes were present in all strains. DNA sequences of *cas* genes were aligned and variations at 15 positions within 8429 bp, i.e. 0.2 % sequence diversity was observed. Evolutionary classification of this CRISPR/*cas* system indicated that it belongs to class 1, type I-F, initially identified in *Yersinia pseudotuberculosis* [57]. The same type of CRISPR/cas system has been reported very recently in *Xanthomonas albilineans* [58].

The spacers of *Xf*-CRR-2 of all *X. fragariae* strains were aligned (Fig. 2a). The alignment allowed splitting of the sequences into two CRISPR groups called *Xf*-CGr-I and *Xf*-CGr-II (*X. fragariae* CRISPR Group). None of the spacers was shared between these groups. In *Xf*-CGr-II, the spacers were conserved among all strains with the exception of the deletion of the eleventh spacer in two Brazilian strains ICMP 5813 and NCPPB 3743. *Xf*-CGr-I showed more evolutionary events, with deletions and variations of spacers. The differential presence or absence of spacers led to the identification of three subgroups: *Xf*-CGr-IA, *Xf*-CGr-IB and *Xf*-CGr-IC (Fig. 2a). Subgroup *Xf*-CGr-IA harbours the most complete spacer sequence most distantly from the leader sequence in *Xf*-CRR-2 and can be thus considered to be the most ancestral set-up. The loss of spacers 3, 6, 7 and 24 and the acquisition of spacers 54 to 60 led to the formation of subgroup *Xf*-CGr-IB, while subgroup *Xf*-CGr-IC, which appears to be the most recent in terms of evolutionary events, resulted from the loss of ancestral spacers 1, 2, 4 and 5 as well as of spacers closer to the leader sequence such as spacers 25–28, 30 and 33–38, and from the acquisition of spacers 77–87 (Fig. 2b). This evolutionary hypothesis relies on the assumption that spacer 85 was acquired independently in both subgroups *Xf*-CGr-IA and *Xf*-CGr-IC, a mechanism that has previously been demonstrated in other bacteria [26]. *Xf*-CGr-IB and -C subgroups harboured CRISPR spacer sequences variability, due to losses or acquisitions of spacers (Fig. 2b). Two different spacer sequences (labelled by A and B; Fig. 2b), were observed in *Xf*-CGr-IB and five spacer sequences were observed in *Xf*-CGr-IC (labelled A to E; Fig. 2b).

Strains belonging to *Xf*-CGr-IA were isolated between 1960 and 1982 in the USA. *Xf*-CGr-IB strains were isolated between 1996 and 2010 in the Netherlands, Belgium and Portugal. Group *Xf*-CGr-IC, which shows the largest diversity from the three subgroups analysed, contains strains isolated between 1995 and 2014. On the other hand, *Xf*-CGr-II strains were isolated between 1966 and 1997. Group *Xf*-CGr-I strains were only isolated in Europe and in the USA, while *Xf*-CGr-II strains were additionally obtained from Brazil, New Zealand and Australia.

The screening of spacer sequences against all *X. fragariae* genomes indicated that sequences corresponding to some spacers were present in genomes of *X. fragariae* in regions annotated as encoding phage-related proteins. Protospacers corresponding to spacers 23, 88, 89 and 90, present in *Xf*-CGr-IB and *Xf*-CGr -IC (Fig. 2; Table S3), were found in the genomes of strains belonging to *Xf*-CGr-II in close proximity to each other at a location annotated as phage-related protein. Similarly, genomes from *Xf*-CGr-IA and *Xf*-CGr-IC harboured sequences corresponding to spacers included in the CRR from *Xf*-CGr-II, (i.e., spacers 206, 207, 212, 213 and 215; Fig. 2; Table S3) in a region containing phage-related proteins. Furthermore, sequences corresponding to spacers 49, 50, 63, 74, and 88–90 (Fig. 2; Table S3) were present in a phage-related region in strains from *Xf*-CGr-IC, which do not include any of these spacers in their CRR. These mutual exclusion events between spacers in the CRR and corresponding protospacers in the genome confirm the role of the CRISPR/*cas* system in preventing invasion by foreign nucleic acid elements, such as phages or plasmids.

### *In silico* VNTR prediction and marker screening for *X*. *fragariae* diversity

A total of 55 tandem repeat regions (TR1 – TR55; Table S4) were identified in the genome of the reference strain *X. fragariae* LMG 25863. VNTR repeat sizes were between 3 and 33 bp with a number of repetitions of between 2.5 and 16. The 58 *X. fragariae* genomes (Table S1) were screened for VNTR presence as well as variability among strains. A total of 19 VNTRs were excluded from the analysis: five for showing no variation among all 58 strains and 14 due to the absence of these markers in most of the genomes (Table S4). The dataset contained thus 36 VNTRs (Table 2), of which 12 were located in intergenic regions. Twenty-four VNTRs were located within predicted genes, these being, among others, related to RNase inhibition, transcriptional regulation or virulence, such as a type III effector (XopF1), two type III secretion proteins (HrpF and HrpW) and a type IV secretion protein VirD4. The variation of length due to the variability of VNTR fragments could therefore affect their gene expression or protein function [59].

**Table 2.** List of 36 VNTRs found in the reference genome of *Xanthomonas fragariae* LMG 25863 (GenBank WGS accession prefix AJRZ01), which were used in the study to discriminate strains

**Locus**	Contig*	**Start** **(bp)**	**End** **(bp)**	**Motif** **(bp)**	Repeats*	**Locus tag**	**Annotated as**†	**Number of repeats**		**Number of alleles**
								**Min.**	**Max.**	
TR01	6	4 772	4 893	14	2.9	Intergenic	NR	1.9	2.9	2
TR02	6	33 485	33 604	13	3	Intergenic	NR	2	3	2
TR04	10	22 939	2 306	6	7	O1K_01649	TonB dependent receptor	6	9	4
TR05	10	57 619	57 727	7	4.1	O1K_01819	RNase III inhibitor	2.1	4.1	3
TR06	10	113 373	113 483	7	4.4	Intergenic	NR	2.4	4.4	3
TR07	11	34 699	34 815	8	4.6	Intergenic	NR	3.6	7.6	5
TR08	11	63 741	63 855	6	5.8	O1K_02551	Type III effector XopF1	3.8	5.8	3
TR09	11	69 814	69 290	6	4.5	O1K_02561	HrpW protein complement	3.5	5.5	4
TR10	11	114 548	114 697	7	10	Intergenic	NR	2	8	4
TR12	15	3 006	3 118	8	4.1	Intergenic	NR	3.1	4.1	2
TR15	20	153 324	153 447	6	7.3	Intergenic	NR	3.3	27.3	13
TR16	22	72 745	72 903	14	5.6	Intergenic	NR	1.6	12.6	6
TR20	23	45 814	45 924	6	5.1	O1K_06782	Transposase IS1478	1.1	6.1	6
TR21	35	2 769	2 931	22	3.7	O1K_07187	Plasmid and phage iteron-binding protein	1.7	3.7	2
TR25	50	325	501	6	16.1	O1K_08577	Hypothetical protein	13.1	20.1	5
TR28	66	3 990	4 116	9	5.2	O1K_09027	Ca^2+^ binding protein	5.2	6.2	2
TR29	66	78 076	78 185	7	4.2	O1K_09387	Relaxation protein	3.2	5.2	3
TR30	66	190 542	190 648	6	4.5	O1K_09987	DeoR family transcriptional regulator	3.5	4.5	2
TR31	66	225 749	225 856	6	4.6	Intergenic	NR	2.6	4.6	3
TR32	66	271 094	271 209	6	6	O1K_10472	Cell-wall-associated hydrolases	3	6	3
TR33	66	373 093	373 213	6	6.8	O1K_10982	Hypothetical protein	4.8	11.8	5
TR34	66	417 716	417 833	15	2.5	O1K_11162	Hypothetical protein	1.5	2.5	2
TR35	76	50 196	50 303	7	4	Intergenic	NR	4	10	4
TR37	76	152 482	152 610	7	7	O1K_12200	Conserved interspecies protein	3	8	6
TR38	76	199 486	199 645	7	11.4	O1K_12380	Ribonucleotide diphosphate reductase subunit beta	2.4	31.4	26
TR39	76	221 288	221 416	5	9.8	Intergenic	NR	3.8	15.8	8
TR41	77	27 938	28 132	12	9.5	O1K_12901	HrpF protein	3.5	10.5	5
TR43	77	29 623	29 735	7	4.7	O1K_12921	Transposase IS1478	3.7	5.7	4
TR44	77	153 680	153 785	5	5.2	O1K_13431	Hypothetical protein	5.2	6.2	2
TR45	77	161 777	161 882	3	8.6	O1K_13486	mRNA 3′ end processing factor exonuclase	3.6	8.6	4
TR47	84	85 036	85 156	6	6.8	O1K_17353	Membrane associated phospholipide phosphatase	2.8	6.8	4
TR49	94	78 449	78 567	14	2.7	O1K_19121	Phasin family protein	1.7	2.7	2
TR51	94	155 369	155 511	24	2.6	O1K_19561	Type IV secretion system protein VirD4	2	3	3
TR52	95	67 136	67 286	8	8.8	Intergenic	NR	4.8	11.8	6
TR54	96	76 525	76 707	33	3.1	O1K_20482	Hypothetical protein	2.8	4.1	3
TR55	96	93 712	93 818	6	4.5	O1K_20532	RsuA pseudouridylate synthase	4.5	6.5	3

*The contig numbers and the repeat number refer to the *X. fragariae* LMG 25863 genome (AJRZ01000000), which was the reference genome used for screening.

†NR, not relevant.

**Fig. 3.**
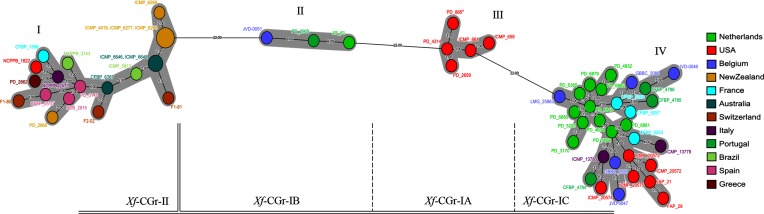
Minimal-spanning tree based on the numbers of repeats per VNTR and per strain (Table S3) as compiled in BioNumerics 7.0. The distances between strains reflect number of different VNTRs per strain and are indicated by numbers on the lines. The colours represent the geographic origin of each strain and the diameter of circle is proportional to the number of strains with the same VNTR genotype. The MLVA types (i.e. I to IV) are indicated above each group of strains. CRISPR types (i.e. *Xf*-CGr) are shown below the tree to emphasize the congruent grouping between CRISPRs and VNTRs methods.

VNTRs showed between two and eight alleles (Table 2), whereas VNTRs number 15 and 38 showed a higher variability with 13 and 26 alleles respectively. Some of the VNTRs showed only two alleles (Table 2), whereas a few VNTRs showed a higher variability with from 10 to 20 different alleles. A minimal-spanning tree revealed that the analysed traits can be used to distinguish the isolates into four distinct groups (Fig. 3). The MLVA groups were defined by the branches where large differences were observed. Indeed, three branches of the MLVA tree were showing differences in over 20 VNTRs out of the 36 markers, whereas most of branches were showing a low variation of between one and six different markers. Although MLVA showed a large intra-group genetic variability, no clear inter-group relationship was explained by the analysis. The composition of the 36 VNTRs allowed the identification of a total of 55 genotypes, and only two genotypes were identical in multiple strains (a first genotype is shared by ICMP 4019, ICMP 6277 and ICMP 6278, while a second one encompasses ICMP 6646 and ICMP 6648; Fig. 3). The VNTR analysis showed congruent strain population structure compared with CRISPR analysis based on the observed strain grouping, therefore CRISPR grouping nomenclature (*Xf*-CGr) was used throughout the whole analysis for clarity purpose.

### Design of a CRISPR-based typing tool for *X. fragariae* group identification

Three primer sets were designed based on spacers of the CRISPR *Xf*-CRR-2 in order to discriminate strains and assign them to the obtained *Xf*-CGr (sub)groups (Table 3). Primer set *Xf*-A was designed based on spacers 1 and 9 (Fig. 2) and generated amplicons for strains of *Xf*-CGr-IA and -IB with a 180 bp difference. This difference in size corresponds to three missing spacers (3, 6 and 7) and their repeats in *Xf*-CGr-IB strains. Primer set *Xf*-B was designed based on spacers 10 and 17 (Fig. 2) and amplified strains of the whole *Xf*-CGr-I. Compared with *Xf*-CGr-IA and -IB, strains from *Xf*-CGr-IC displayed a 60 bp smaller amplicon due to the absence of spacer 13 and its direct repeat in those strains. Finally, primer set *Xf*-C was designed based on spacers 208 and 213 (Fig. 2), and amplified strains of *Xf*-CGr-II. Two strains (ICMP 5813 and NCPPB 3743) of this group do not harbour spacer 210 and therefore a 60 bp reduction of the amplicon size was observed.

**Table 3.** List of primer sets designed in order to assign isolates to one of the four CRISPR groups (*Xf*-CGr-IA, -IB, -IC and -II) defined in this study The predicted sizes of the amplicons were based on the genome information of *Xanthomonas fragariae* LMG 25863 (GenBank WGS accession prefix AJRZ01), but also on other strain information relating to the different groups in order to assess the fragment sizes per group. The spacer location indicates which spacers are included in the amplified fragments.

**Primer set name**	**Primer name**	**Spacer location**	**Sequence (5′–3′)**	Expected amplicon size per group (bp)*			
				**IA**	**IB**	**IC**	**II**
*Xf*-A	A-Forward	Spacer 1	ACT CAT CCA GAG CTT CAG TAG	514	334	na	na
	A-Reverse	Spacer 9	TCT ATG GGG AAA TCA TTT TCG				
*Xf*-B	B-Forward	Spacer 17	CGC ACC CTT GCA GAC TGT A	443	443	383	na
	B-Reverse	Spacer 10	CCT GAC TTC TGC AAT CAG GGC				
*Xf*-C	C-Forward	Spacer 208	GGT GCA TCG CGC TTT GTT TTC T	na	na	na	255/315†
	C-Reverse	Spacer 213	GAA GAT CGC CAG GAG GAC CAG G				

*NA, no amplicon.

†For strains within *Xf*-CGr-II, the number of CRISPR spacers varied between 16 and 17.

**Fig. 4.**
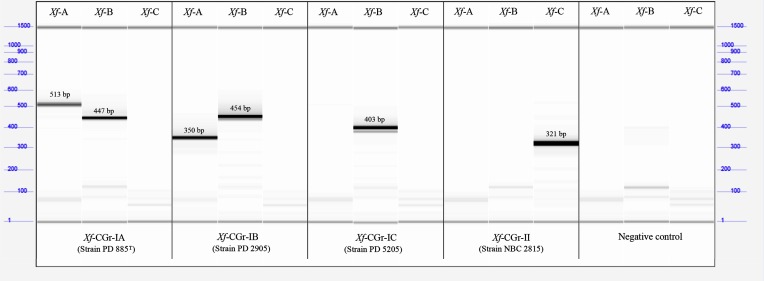
PCR amplicons obtained with primers sets *Xf*-A, *Xf*-B and *Xf*-C (Table 3) on a set of four strains belonging to the four different described CRISPR subgroups. PCR products were analysed with high-resolution gel electrophoresis, which adds internal markers of 1 bp and of 1500 bp. This serves as reference to calculate the length of the amplification fragments per *Xf*-CGr (sub)group.

The combination of three sets of primers allows the assignment of a random *X. fragariae* isolate to the respective CRISPR group. PCR products of four samples belonging to the four described (sub)groups (PD 885^T^, PD 2905, PD 5205 and NBC 2815) were used as reference for band sizes and presence or absence per primer set using high-resolution gel electrophoresis (Fig. 4). The genotypes of all strains used in this study were confirmed with these three primer sets and visualized on an agarose gel (Fig. S2). The amplicon size obtained per isolate corresponded to the pattern predicted from the genome sequence and allowed these strains to be assigned to their predicted *Xf*-CGr groups.

## Discussion

In this study, a representative collection of strains of *X. fragariae* with miscellaneous geographic origins and years of isolations was analysed. Whole-genome sequencing was used in order to investigate the diversity and to access all molecular markers at once. Two molecular markers were chosen on the sequenced *X. fragariae* genomes for evolutionary tracking purposes, allowing a higher discriminatory resolution than previous methods, which included RFLP, DNA–DNA hybridization, fatty acid profiling, REP-PCR or AFLP [37, 43]. These methods resulted in discordant strain grouping and were applied to strains from limited geographic origins. CRISPR analysis could be exploited to infer a chronological perspective to the obtained strain variability [20, 21], thanks to a representation of diverse chronological and geographic origins. Although the different genome sequencing technologies applied in this study yielded genomes with variable assembly quality, the required markers provided sufficient information to reconstruct the population structure of the *X. fragariae* strains.

The genetic diversity of the *X. fragariae* isolates was assessed with four types of genetic markers: ANIb, housekeeping genes (MLSA), CRISPRs and VNTRs (MLVA). The methods gave congruent results but differed in their discriminative potential. ANIb confirmed the high similarity of the isolates and confirmed that only one bacterial species was found. MLSA could only separate the isolates into two groups of strains with no resolution within the groups. CRISPR analysis resolved the same two groups as MLSA. None of the CRISPR spacers was shared between *Xf*-CGr-I and *Xf*-CGr-II, indicating that separation between the two groups probably took place before the first recognized *X. fragariae* isolate was identified. The results of CRISPR spacer analysis indicated a higher diversity within group *Xf*-CGr-I and could be used to separate the *Xf*-CGr-I isolates into three subgroups. Finally, MLVA resulted in a total of four clearly separated groups that corresponded to the four groups identified by CRISPR typing, but could resolve most isolates to the strain level. CRISPRs and MLVA results were complementary and provided a robust overview of the population structure of *X. fragariae*. The combination of the two high-resolution molecular markers, CRISPRs and MLVA, was used to define the population structure of another plant bacterial disease, *Erwinia amylorova* [2, 26], for which a large number of strains were available. A good concordance between phylogenies of CRISPRs and MLVA was observed, and MLVA allowed a deeper phylogeny than CRISPRs [2, 26]. In a similar comparative analysis of subtyping methods for *Salmonella enterica* [60], results of the latter study indicated that CRISPR analysis could delineate major lineages between strains, whereas MLVA could not reveal such patterns.

Based on the CRISPR repeat regions [61], an evaluation of the chronological succession of the different groups was attempted. This approach indicated that the earliest isolated strain of *X. fragariae*, strain PD 885^T^ [35], was, evolutionarily, among the most ancient according to the results of the CRISPR analysis. Given the presence of the most ancestral spacers in strains of *Xf*-CGr-IA, this subgroup can be considered as closest to the evolutionary ancestor. The subgroups *Xf*-CGr-IB and *Xf*-CGr-IC are then more recent and resulted from complex evolution following spacer deletions and acquisition of novel spacers. The CRISPR sequence of *Xf*-CGr-II did not change significantly between the first isolated strain in 1966 and the latest isolate in 1997.

The genetic relationship between the main CRISPR groups remains unclear, as none of the CRISPR spacers was shared between the two groups, making any direct comparison impossible. Nevertheless, some spacers located within the CRR of a given *Xf*-CGr were present as protospacers in phage-related regions of the other *Xf*-CGr and vice versa. This indicated that the two groups were facing a similar phage background. One group may have integrated the phages in the genome, whereas the other group acquired new spacers targeting their sequences, thus effectively protecting them from subsequent invasion attempts.

VNTRs are considered to be a ‘fast molecular clock’ [1]. The fast evolution of the repeats can explain the variability within groups and subgroups among strains for the general picture over the 36 markers that were usable in this study. High intra-group variability was observed using MLVA analysis, but the number of available isolates was too low to allow conclusions to be drawn about evolutionary patterns. The heterogeneous geographic origins of strains within (sub)groups indicated that exchanges, probably due to movement of infected plant material, between continents were common and evolution was not directed separately between continents.

CRR variation was mainly observed in *Xf*-CGr-I, and a non-linear evolution seemed to have taken place as various events seemed to have happened independently and resulted in a branching pattern of evolution. No clear pathways of dissemination between geographic locations could be defined. *Xf*-CGr-I has not been reported to be present on the Southern hemisphere, even though strains were intercepted at the border before they entered New Zealand from the USA. Strains from group *Xf*-CGr-II have been isolated from broad geographical origins: Europe, South America, North America and Oceania.

Between 1994 and 2011, 61 interceptions of infected material intended to be sent to another country were reported [62], which confirms the continuous worldwide spread of this bacterial disease. The probability of bacterial dispersal from a third country by plant material trade is therefore considered to be very high and worldwide. In 2014, some of the *X. fragariae* strains included in this study (ICMP 50572, ICMP 50573, ICMP 50574 and ICMP 50575), were intercepted on strawberry plants arriving from the USA at the border of New Zealand, where the pathogen is considered to be eradicated (https://gd.eppo.int/taxon/XANTFR/distribution). In Australia, three distinct outbreaks have taken place: in 1975 in New South Wales [63], in 1994 in South Australia [64] and more recently in 2010 in Queensland [65]. After each outbreak, the disease was considered eradicated after control measures [66]. It is considered unlikely that the same bacteria caused the outbreaks, as genomic fingerprinting with REP-PCR indicated that the strains were genetically distinct from strains isolated during the previous outbreaks [64, 65]. These are concrete examples showing that infected plant material can be transported through country borders. The risks of introductions into new geographic areas is high as *X. fragariae* can be present asymptomatically in planting material for a long period [67]. The disease has been reported recently in Mexico, Iran and China [68–70], but we were not able to access the strains. Access to a strain is crucial to understand how the pathogen is transferred between countries. To prevent further spread of the disease, highly specific and effective methods have been designed for detection of *X. fragariae* [44, 71, 72].

An assay based on three PCR primer sets was developed to assign novel isolates to the CRISPR (sub)groups detected in this study. The assay was designed to be suitable for use in standard plant pathology laboratories, since it provides the opportunity to perform strain typing without the requirement for whole-genome sequencing. The access to typing schemes of newly reported and upcoming strains would be essential to refine the analysis. Once a sufficient number of isolates from geographically diverse origins of isolation is reached, four open questions may be answered. The first is whether isolates from both groups are co-existing or if only one group persists nowadays. The second might require full genomic information, including access to full VNTR and CRISPR sequences, in order to be able to reconstruct how *X. fragariae* is spread geographically through plant material movements. As *Xf*-CGr-I and -II were separated before the description of the species, the third question may require a deeper analysis of genome sequences in order to determine how fast strains evolved in order to estimate when the separation of the groups has occurred. Finally, the fourth question may rely on finding a common isolate that can be classified as common ancestor of the two main CRISPR groups.

Due to the close similarities between isolates of *X. fragariae*, only few strains have been deposited in bacterial culture collections. Considering the small amount of strains available, the obtained diversity with both VNTRs and CRISPRs could highlight a higher-than-expected variability. Superior typing methods now allow discrimination of strains with a higher resolution compared with previous studies. This outcome may allow the enhanced monitoring of the population structure, especially in the case of a new outbreak of the pathogen. Deeper comparative genomic analyses as well as pathogenicity tests are required to further characterize the genetic and phenotypic difference between the obtained CRISPR-based groups.

## Data bibliography

1. All genome sequencing data have been deposited in EMBL under the study code PRJEB25730 (https://www.ebi.ac.uk/ena/data/view/PRJEB25730) with the accession numbers ERR2528852–ERR2528906 for raw reads (https://www.ebi.ac.uk/ena/data/view/ERR2528852-ERR2528906) and ORXX01000000–ORZZ01000000 for genome assemblies (https://www.ebi.ac.uk/ena/data/view/ORXX01000000-ORZZ01000000).

## Supplementary Data

Supplementary File 3

## Supplementary Data

Supplementary File 4
