# Supplementary material for: Genome-based population structure analysis of the strawberry plant pathogen Xanthomonas fragariae reveals two distinct groups that evolved independently before its species description
Source: Microb Genom. 2018 Jun 6;4(7):e000189. doi: 10.1099/mgen.0.000189 (PMC6113873; doi:10.1099/mgen.0.000189)
Supplement: Supplementary File 3 [file mgen-4-189-s001.pdf]

MICROBIAL GENOMICS

Research paper

## SUPPLEMENTAL INFORMATION belonging to:

# Genome-based population structure analysis of the strawberry plant pathogen *Xanthomonas fragariae* reveals two distinct groups that evolved independently before its species description

Michael Gétaz,* Marjon Krijger,† Fabio Rezzonico,* Theo H.M. Smits,* Jan M. van der Wolf,† Joël F. Pothier*‡

* *Environmental Genomics and Systems Biology Research Group, Institute of Natural Resource Sciences, Zurich University of Applied Sciences (ZHAW), CH-8820 Wädenswil, Switzerland.*

† *Wageningen University & Research, Wageningen, the Netherlands.*

---

## SUPPLEMENTARY TABLES

**Table S1.** List of *Xanthomonas fragariae* strains used in this study with source, geographic origin, year of isolation, MLVA and CRISPR groups, sequencing method and assembly metrics. *(Excel file; 000189_2.xlsx)*

**Table S2.** Average Nucleotide Identities based on BLAST (ANIb) results in percentage calculated on the 58 *Xanthomonas fragariae* genomes using PYANI v.0.2.0. Values were ranging between 99.48% and 99.97% identity. *(Excel file; 000189_2.xlsx)*

**Table S3.** List of the 109 CRISPRs found among all the 58 *Xanthomonas fragariae* strains analyzed in this study. The spacers are aligned according to the *Xf*-CGr obtained in this study.

The sequence of each spacer is provided and the spacer composition per strain is expressed with colored block containing the number of its spacer. Spacers were numbered from 1 to 93 and from 200 to 216 for *Xf*-CGr-I and *Xf*-CGr-II, respectively. The black block at the bottom of the table indicates the leader sequence, which is the region of incorporation of new spacers in the CRISPR. *(Excel file; 000189_2.xlsx)*

**Table S4.** VNTRs used in this study. The 55 VNTRs initially detected in *Xanthomonas fragariae* LMG 25863 are reported in this table. The detected number of repeats for each of these 55 VNTRs is reported for every strain used in this study. Only the 36 VNTRs at the top were used for the analyse while the 19 last ones were not used due to lack of information in some strains or identical repeat number for all strains. The table provides also the sequence of the repeats, the number of repeats and the start/end positions (concatenated version of the genome) of the VNTRs found in the genome of *X. fragariae* LMG 25863. For VNTRs located in a coding region, the annotation present at this position in the genome of *X. fragariae* LMG 25863 is also reported. *(Excel file; 000189_2.xlsx)*

---

## SUPPLEMENTARY FIGURES

**Fig. S1.** Phylogenetic tree resulting from an MLSA of seven partial housekeeping genes (*atpD*, *dnaK*, *etp*, *fyuA*, *glnA*, *gyrB* and *rpoD*) on *Xanthomonas fragariae* strains used in this study. Concatenated sequences (~6 200 bp) were analysed with Maximum Likelihood with 1 000 bootstraps. Two groups (*Xf*-CGr-I and *Xf*-CGr-II) are highlighted in addition of a *Xanthomonas arboricola* pv. *fragariae* strain (LMG 19146) used as outgroup and for which distance to *X. fragariae* strains was already assessed previously (Vandroemme *et al*. 2013). The bootstrap values in percentages are represented on the tree close to the nodes. (*This pdf file; 000189_1.pdf*)

**Fig. S2.** *Xf*-CRISPR PCR results performed on 56 *Xanthomonas fragariae* strains using *Xf*-A, *Xf*-B and *Xf*-C primers sets. (a) With the *Xf*-A primer set, an amplicon is observed for both *Xf*-CGr-IA and *Xf*-CGr-IB isolates at 514 bp and 334 bp, respectively. This set allows thus to distinguish between *Xf*-CGr-IA and *Xf*-CGr-IB isolates (b) The set *Xf*-B produces an amplicon with all *Xf*-CGr-IA, *Xf*-CGr-IB and *Xf*-CGr-IC isolates of either 443 bp (*Xf*-CGr-IA and *Xf*-CGr-IB) or 383 bp (*Xf*-CGr-IC). This set allows thus to distinguish *Xf*-CGr-IC from *Xf*-CGr-IA and *Xf*-CGr-IB isolates. (c) The set *Xf*-C gives only an amplicon with *Xf*-CGr-II isolates for which the size observed is either 315 bp (*Xf*-CGr-II-A) or 255 bp (*Xf*-CGr-II-B). This set allows thus to distinguish *Xf*-CGr-II isolates but also allows to differentiate *Xf*-CGr-II-A and *Xf*-CGr-II-B isolates due to the absence of a single spacer in this latter group leading to a decrease of 60 bp. (*This pdf file; 000189_1.pdf*)

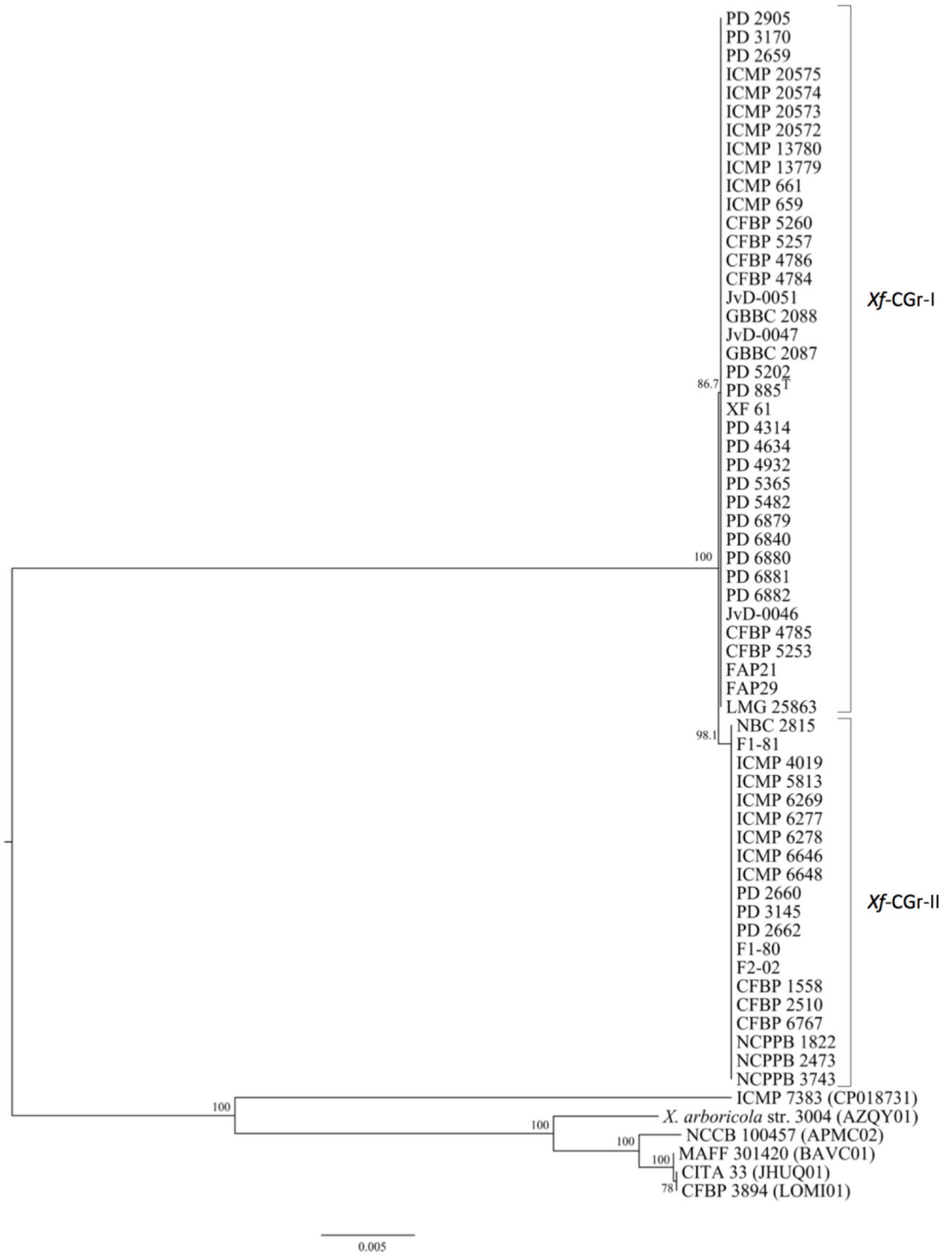


**Fig. S1.** Phylogenetic tree resulting from an MLSA of seven partial housekeeping genes (*atpD*, *dnaK*, *etp*, *fyuA*, *glnA*, *gyrB* and *rpoD*) on *Xanthomonas fragariae* strains used in this study. Concatenated sequences (~6 200 bp) were analysed with Maximum Likelihood with 1 000 bootstraps. Two groups (*Xf*-CGr-I and *Xf*-CGr-II) are highlighted in addition of a

*Xanthomonas arboricola* pv. *fragariae* strain (LMG 19146) used as outgroup and for which distance to *X. fragariae* strains was already assessed previously (Vandroemme *et al*. 2013). The bootstrap values in percentages are represented on the tree close to the nodes.

(a)

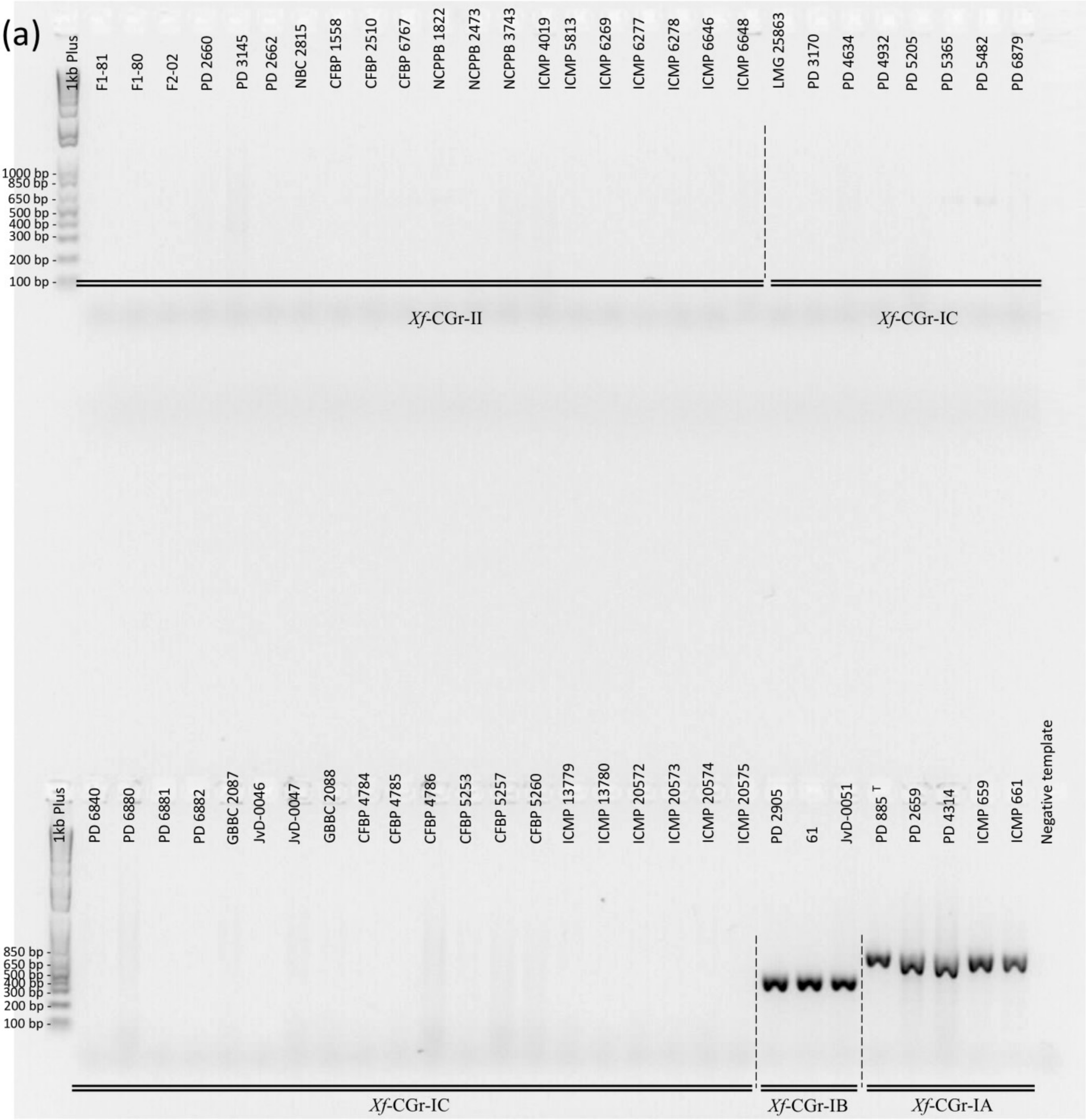
1kb Plus
F1-81
F1-80
F2-02
PD 2660
PD 3145
PD 2662
NBC 2815
CFBP 1558
CFBP 2510
CFBP 6767
NCPPB 1822
NCPPB 2473
NCPPB 3743
ICMP 4019
ICMP 5813
ICMP 6269
ICMP 6277
ICMP 6278
ICMP 6646
ICMP 6648
LMG 25863
PD 3170
PD 4634
PD 4932
PD 5205
PD 5365
PD 5482
PD 6879
1000 bp -
850 bp -
650 bp -
500 bp -
400 bp -
300 bp -
200 bp -
100 bp -
Xf-CGr-II
Xf-CGr-IC
1kb Plus
PD 6840
PD 6880
PD 6881
PD 6882
GBBC 2087
JvD-0046
JvD-0047
GBBC 2088
CFBP 4784
CFBP 4785
CFBP 4786
CFBP 5253
CFBP 5257
CFBP 5260
ICMP 13779
ICMP 13780
ICMP 20572
ICMP 20573
ICMP 20574
ICMP 20575
PD 2905
61
JvD-0051
PD 885 T
PD 2659
PD 4314
ICMP 659
ICMP 661
Negative template
850 bp -
650 bp -
500 bp -
400 bp -
300 bp -
200 bp -
100 bp -
Xf-CGr-IC
Xf-CGr-IB
Xf-CGr-IA

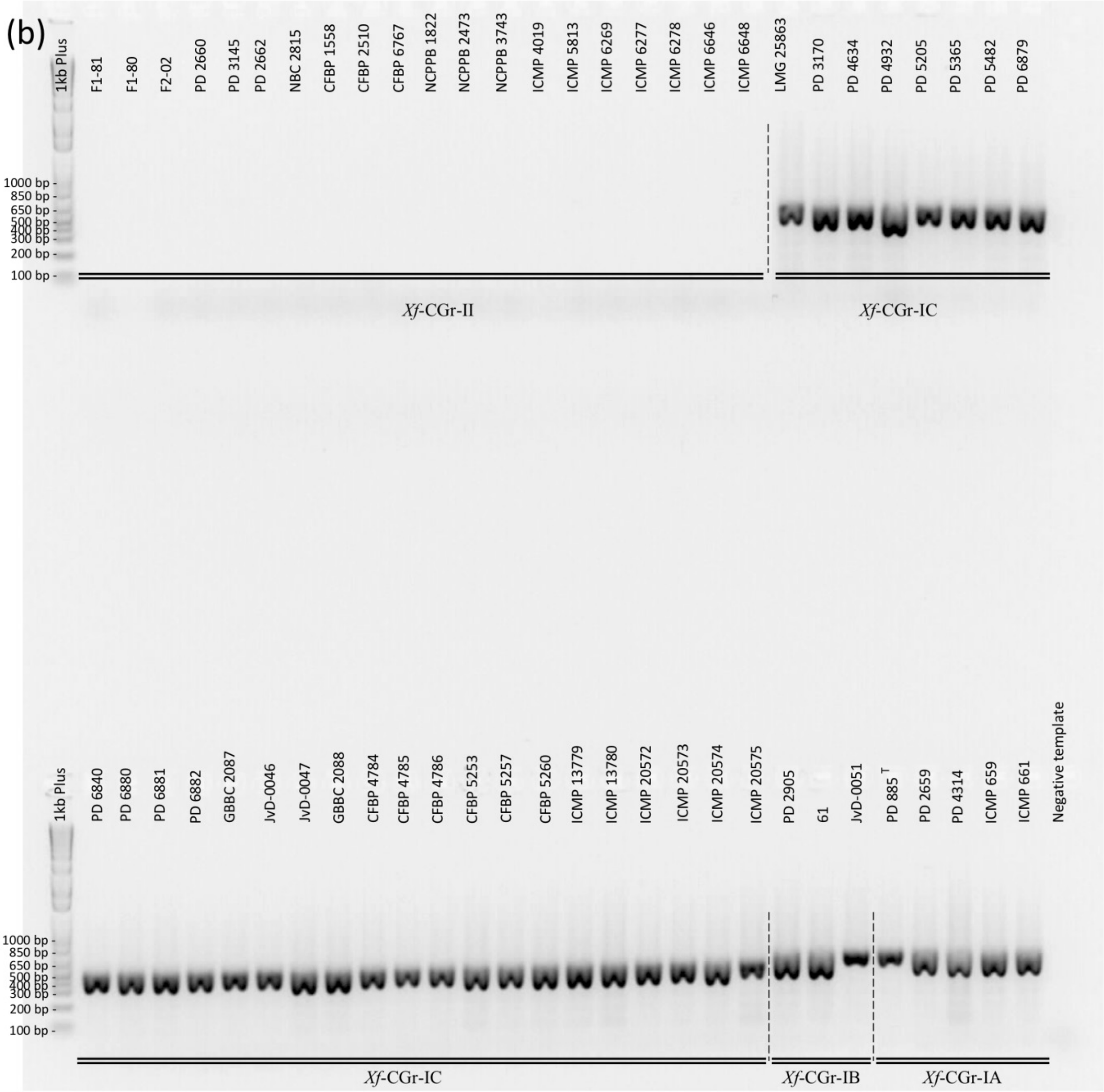
(b)
1kb Plus
F1-81
F1-80
F2-02
PD 2660
PD 3145
PD 2662
NBC 2815
CFBP 1558
CFBP 2510
CFBP 6767
NCPPB 1822
NCPPB 2473
NCPPB 3743
ICMP 4019
ICMP 5813
ICMP 6269
ICMP 6277
ICMP 6278
ICMP 6646
ICMP 6648
LMG 25863
PD 3170
PD 4634
PD 4932
PD 5205
PD 5365
PD 5482
PD 6879
1000 bp
850 bp
650 bp
500 bp
400 bp
300 bp
200 bp
100 bp
Xf-CGr-II
Xf-CGr-IC
1kb Plus
PD 6840
PD 6880
PD 6881
PD 6882
GBBC 2087
JvD-0046
JvD-0047
GBBC 2088
CFBP 4784
CFBP 4785
CFBP 4786
CFBP 5253
CFBP 5257
CFBP 5260
ICMP 13779
ICMP 13780
ICMP 20572
ICMP 20573
ICMP 20574
ICMP 20575
PD 2905
61
JvD-0051
PD 885 T
PD 2659
PD 4314
ICMP 659
ICMP 661
Negative template
1000 bp
850 bp
650 bp
500 bp
400 bp
300 bp
200 bp
100 bp
Xf-CGr-IC
Xf-CGr-IB
Xf-CGr-IA

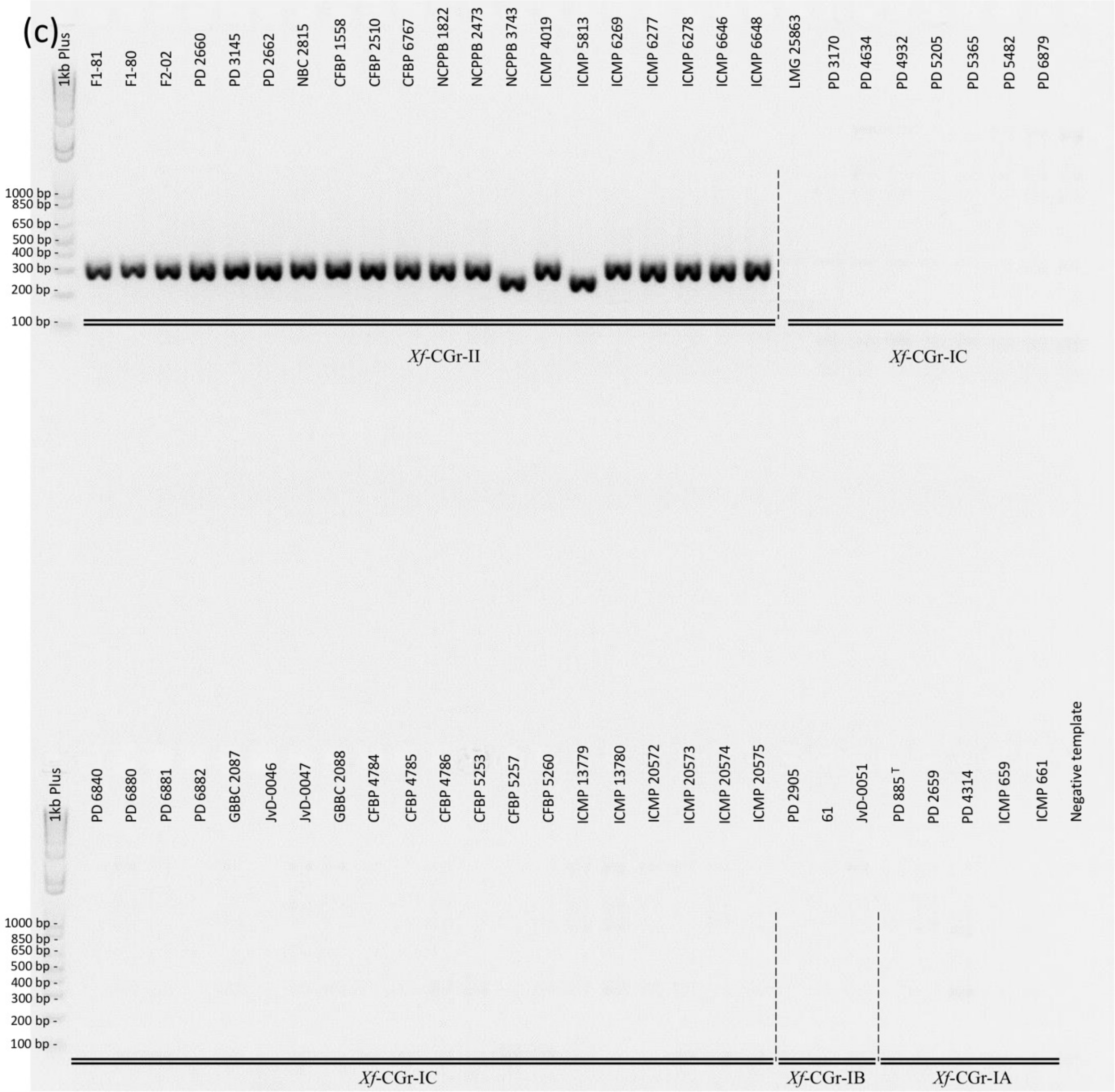


**Fig. S2.** *Xf*-CRISPR PCR results performed on 56 *Xanthomonas fragariae* strains using *Xf*-A, *Xf*-B and *Xf*-C primers sets. (a) With the *Xf*-A primer set, an amplicon is observed for both *Xf*-CGr-IA and *Xf*-CGr-IB isolates at 514 bp and 334 bp, respectively. This set allows thus to distinguish between *Xf*-CGr-IA and *Xf*-CGr-IB isolates (b) The set *Xf*-B produces an amplicon with all *Xf*-CGr-IA, *Xf*-CGr-IB and *Xf*-CGr-IC isolates of either 443 bp (*Xf*-CGr-IA and *Xf*-CGr-IB) or 383 bp (*Xf*-CGr-IC). This set allows thus to distinguish *Xf*-CGr-IC from *Xf*-CGr-IA and *Xf*-CGr-IB isolates. (c) The set *Xf*-C gives only an amplicon with *Xf*-CGr-II isolates for which the size observed is either 315 bp (*Xf*-CGr-II-A) or 255 bp (*Xf*-CGr-II-B).

This set allows thus to distinguish *Xf*-CGr-II isolates but also allows to differentiate *Xf*-CGr-II-A and *Xf*-CGr-II-B isolates due to the absence of a single spacer in this latter group leading to a decrease of 60 bp.
